# Vaccine Effectiveness against DS-1–Like Rotavirus Strains

**DOI:** 10.3201/eid2601.191377

**Published:** 2020-01

**Authors:** Toyoko Nakagomi

**Affiliations:** Retired from Nagasaki University, Nagasaki, Japan

**Keywords:** rotavirus, vaccine effectiveness, DS-1–like backbone genes, viruses, Malawi, children

**To the Editor:** Global emergence of reassortant rotavirus A (RVA) strains possessing the DS-1 backbone genes, such as DS-1–like G1P[8] strains (*1*–*3*), raises 2 key questions of public health importance. First, is the G1P[8] monovalent vaccine (RV1) effective against the DS-1–like G1P[8] strain? Second, does this strain cause more severe disease than the Wa-like G1P[8] strain? 

For the first question, Jere et al. (*1*) showed that RV1 was highly effective against DS-1–like G1P[8] RVAs (vaccine effectiveness [VE] 85.6% [95% CI 34.4%–96.8%]). For the second question, we conducted a study in Hanoi, Vietnam, during the 2012–2013 rotavirus season in which 20 DS-1–like G1P[8] RVAs co-circulated with 49 Wa-like G1P[8] RVAs and 50 G2P[4] RVAs among children <2 years of age who had diarrhea. We found no evidence of increased virulence of DS-1–like G1P[8] strains as measured by Vesikari’s severity scores (*3*). 

However, Jere et al. (*1*) were unable to demonstrate statistically significant VE against DS-1–like G2P[4] or Wa-like G1P[8] strains and were concerned about the low point estimate of the VE against heterotypic G2P[4] strains. Using the previous dataset (*3*), we conducted a post hoc logistic regression analysis to compare any RV1 vaccination status versus 0-dose RV1 vaccination status between strain-specific rotavirus diarrhea case-patients and 127 acute respiratory infection control patients whose RV1 coverage was 49.6%. We calculated VE against DS-1–like G1P[8] as 72.5% (95% CI 10.8%–91.5%; p = 0.045), VE against Wa-like G1P[8] as 90.8% (95% CI 72.9%–96.9%), and VE against G2P[4] as 78.1% (95% CI 49.1%–90.6%). These findings confirm the effectiveness of RV1 against fully heterotypic G2P[4] strains, as shown elsewhere (*4*,*5*), and supplement the study by Jere et al. (*1*) in helping dismiss concern about continued use of the monovalent vaccine, even in places where RVAs with the DS-1 backbone are not uncommon.
